# Measuring the dynamic photosynthome

**DOI:** 10.1093/aob/mcy087

**Published:** 2018-06-04

**Authors:** Erik H Murchie, Shawn Kefauver, Jose Luis Araus, Onno Muller, Uwe Rascher, Pádraic J Flood, Tracy Lawson

**Affiliations:** 1 Division of Plant and Crop Science, School of Biosciences, University of Nottingham, Sutton Bonington, UK; 2 Section of Plant Physiology, Faculty of Biology, University of Barcelona, Barcelona, Spain; 3 Institute of Bio-and Geosciences, IBG-2: Plant Sciences, Forschungszentrum Jülich GmbH, Jülich, Germany; 4 Max Planck Institute for Plant Breeding Research, Carl-Von-Linne-Weg, Köln, Germany; 5 School of Biological Sciences, University of Essex, Colchester, UK

**Keywords:** Photosynthesis, dynamic, steady state, genetics, yield, phenomics

## Abstract

**Background:**

Photosynthesis underpins plant productivity and yet is notoriously sensitive to small changes in environmental conditions, meaning that quantitation in nature across different time scales is not straightforward. The ‘dynamic’ changes in photosynthesis (i.e. the kinetics of the various reactions of photosynthesis in response to environmental shifts) are now known to be important in driving crop yield.

**Scope:**

It is known that photosynthesis does not respond in a timely manner, and even a small temporal ‘mismatch’ between a change in the environment and the appropriate response of photosynthesis toward optimality can result in a fall in productivity. Yet the most commonly measured parameters are still made at steady state or a temporary steady state (including those for crop breeding purposes), meaning that new photosynthetic traits remain undiscovered.

**Conclusions:**

There is a great need to understand photosynthesis dynamics from a mechanistic and biological viewpoint especially when applied to the field of ‘phenomics’ which typically uses large genetically diverse populations of plants. Despite huge advances in measurement technology in recent years, it is still unclear whether we possess the capability of capturing and describing the physiologically relevant dynamic features of field photosynthesis in sufficient detail. Such traits are highly complex, hence we dub this the ‘photosynthome’. This review sets out the state of play and describes some approaches that could be made to address this challenge with reference to the relevant biological processes involved.

## INTRODUCTION

**Fig. 1.**
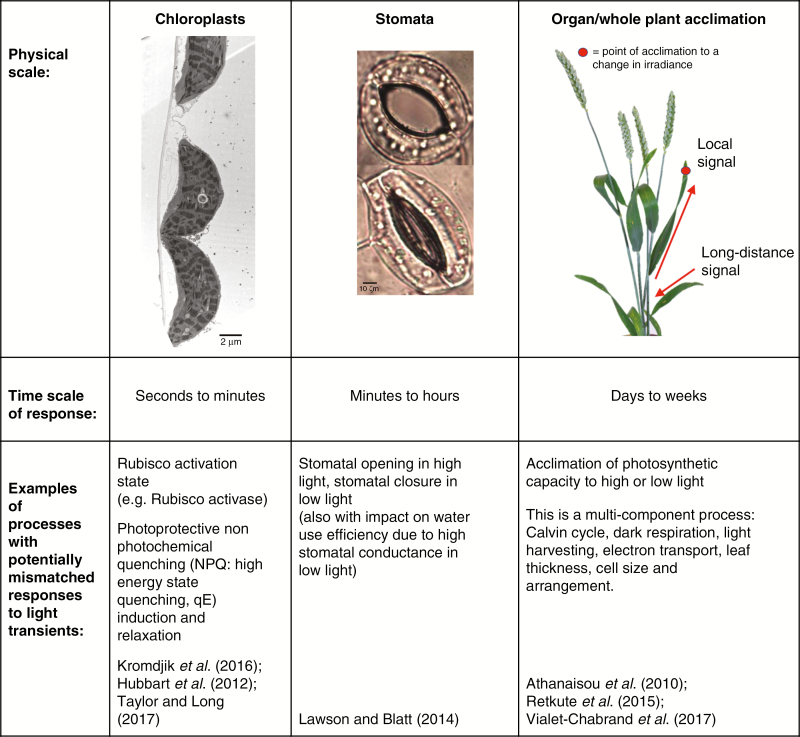
Aspects of dynamic photosynthesis and the prospects for improvement. Steady-state (or temporary steady-state) photosynthesis is easily measured but hard to relate to biomass and yield production. Here we highlight examples of important dynamic processes at different scales that are not necessarily or easily predictable from a steady-state measurement. The purpose is to demonstrate that dynamic processes which influence photosynthetic rates do not necessarily need to occur within seconds or minutes (such as photosynthetic induction) but can also include acclimation which is a process occurring over days or weeks. We have not provided a complete reference list but merely good relevant examples. Image sources (permission obtained): stomata, Kecheli Batta (University of Essex) and chloroplasts of *Monstera deliciosa*, O. Muller (Jülich). Scale bar for chloroplasts and stomata = 2 μm and 10 μm, respectively.

Photosynthesis is perhaps the most studied physiological process in plant science. This is unsurprising given its key role for the energy budget of both plants and the planet. Despite its importance, descriptions of this process often fail to account adequately for the dynamic range with which photosynthesis interacts with its environment. It is a mistake to think of photosynthesis as a single linear process. First, photosynthesis must integrate major primary processes within the plant such as light harvesting, electron transport, photorespiration, gas exchange, and sucrose and starch synthesis and export (Paul and Foyer, 2001; Smith and Stitt, 2007). Control and integration of these primary processes does not occur in isolation as photosynthesis is sensitive to events at the whole-plant level, either by local signalling or via systemic long-range signals (Yano and Terashima, 2001; Coupe *et al.*, 2006; Lee *et al.*, 2016). Secondly, photosynthesis (and counterpart processes such as respiration) is highly responsive to fluctuations in environmental conditions. For example, leaves are subjected to both spatial and temporal gradients in light due to changes in sun angle, clouds passing overhead, and overlapping and moving leaves. Such fluctuations within a canopy can be extremely complex. Indeed, we are beginning to understand that the way in which photosynthesis is regulated in response to fluctuations in the environment is perhaps a more important determinant of plant productivity than its performance under steady-state or temporarily steady-state conditions (Athanasiou *et al.*, 2010; Johnson *et al.*, 2015; Kaiser *et al*., 2015, 2018; Ort *et al.*, 2015; Kromdijk *et al.*, 2016; McAusland *et al.*, 2016; Vialet-Chabrand *et al.*, 2017; Townsend *et al.*, 2018). Here steady state is assumed to include any measurement in which the leaf or plant is temporarily held at a particular set of constant conditions. Measuring the properties of a plant under steady-state conditions is important (and convenient), but it does not always allow a prediction of how that plant will respond in a complex field environment (Poorter *et al.*, 2016; Vialet-Chabrand *et al.*, 2017; Matthews *et al.*, 2018). When photosynthesis does not ‘track’ variations in the environment accurately over time scales of seconds, minutes or days, this can lead to a lowering of resource use efficiency (McAusland *et al.*, 2016) (Fig. 1). Here we use the term ‘photosynthome’ to refer to a set of characteristics that include both the static properties and dynamic responses of the photosynthetic apparatus. A simple example would be the inclusion not only of the light-saturated rate of photosynthesis under a particular set of conditions but also the time taken to reach that rate.

The inclusion of dynamic responses, such as Rubisco activation, photoprotection or stomatal responses, is important as they are not always deducible from the steady-state properties and we do not know which process(es) is (are) limiting under fluctuating conditions. This issue has recently been highlighted using evidence that it is possible to alter genetically the dynamics of key processes such as photoprotection to produce a change in overall plant productivity (Kromdijk *et al.*, 2016). Indeed, it is particularly important that light is accurately tracked by the plant for optimal photosynthetic performance (Sinclair and Muchow, 1999; Mott and Woodrow, 2000; Zhu *et al.*, 2004; Lawson *et al.*, 2012; Murchie and Reynolds, 2012; Lawson and Blatt, 2014; Burgess *et al.*, 2015).

The range of environments in which photosynthetic organisms occur exhibits wide variation in the temporal flux of many environmental parameters, in terms not only of light, but also of factors such as temperature and humidity. Plants have numerous mechanisms for adjustment to such environments; some of the most extreme instances are high-altitude equatorial environments that have an intensely diurnal climate, namely winter every night and summer every day. Whilst morphological adaptations to such extreme temperature fluctuations are well documented, the physiological adjustments are not (Hedberg, 1970). Natural genetic variation for the dynamic properties of photosynthesis is poorly documented despite recent studies that show considerable promise for increasing crop yields not only *per se* but also in the face of increasingly unpredictable climates (Ray *et al.*, 2013; Kromdijk *et al.*, 2016).

The high-throughput measurement of plant phenotypes (phenomics) is a broad term that refers to quantification of plant form and function (and component processes) at the whole-plant level. It has received much attention recently due to the rapid expansion in technology and applications for sensing plant growth and plant processes, and the increasing need to assess large numbers of plants at speed (e.g. see recent reviews by Tester and Langridge, 2010; Furbank and Tester, 2011; Pieruschka and Poorter, 2012; Dhondt *et al.*, 2013; Araus and Cairns, 2014; Flood *et al.*, 2016; Hawkesford and Lorence, 2017). For the purposes of crop improvement, this is critical because genotypic diversity needs to be rapidly linked to phenotypic diversity to inform marker-assisted selection. Typically, conventional breeding takes several years or more so, and thus rapid and high-resolution phenotyping is essential to leverage the power of the genomics revolution and drive through the production of new varieties with beneficial traits on a time scale that permits adaptation to current climate change (Challinor *et al.*, 2016).

Photosynthesis is now established as an important target for improving yield, largely due to its effects on overall canopy radiation use efficiency (the amount of biomass produced per unit radiation intercepted) (Long *et al.*, 2006; Zhu *et al*., 2008, 2010; Murchie *et al.*, 2009; Flood *et al.*, 2011; Hubbart *et al.*, 2018). Hence crop phenotyping must incorporate measurements of plant photosynthesis (Pieruschka and Poorter, 2012; Murchie and Lawson, 2013). However, the importance of the dynamic responses of photosynthesis raises a key problem that has not been adequately addressed: it is difficult to capture photosynthetic responses within (rapidly) fluctuating environments, especially in the field. This is a challenge which must be met because field phenotyping is essential to allow plants to ‘express’ the appropriate phenotype, something that is not always possible in controlled environments, even glasshouses (Poorter *et al*., 2012, 2016; Vialet-Chabrand *et al*., 2016). We are at a point where we require a revolution in technology and methodology for measuring photosynthesis at wide spatial (leaf, 3-D canopy, field) and temporal scales in order to capture responses that are relevant to both agricultural productivity and ecosystem health. This review assesses the current strategies for quantifying (phenotyping) photosynthesis over such scales, focusing on the need to measure dynamic responses to the environment meaningfully. In this review, responses to light fluctuations receive emphasis. This is due to the high sensitivity of the photosynthetic process to light over short time scales, the limitation to crop yield by canopy radiation use efficiency and the substantial fluctuations of light in nature which have many stochastic components.

## NATURAL GENETIC VARIATION IN PHOTOSYNTHESIS

Phenomic technologies fundamentally depend on having relevant germplasm available. A major component of the epistemic value of high-throughput measurements is in providing empirical data by which the genotype–phenotype map can be resolved for a given population. The choice of germplasm is a key consideration in any research programme as it will determine the nature of the insights the high-throughput phenotyping is likely to generate. For example, a population of plants derived from a mutation experiment will mostly provide insight into loss of function and identify key, often highly conserved, genes involved in the phenotype; on the other hand, a collection of accessions derived from the wild may give insight into gain of function or adaptive differences, particularly if the accessions were collected across an environmental gradient, such as temperature or precipitation (Flood *et al.*, 2011; Hancock *et al.*, 2011). Photosynthesis is an intrinsically dynamic trait which exhibits a high degree of environmental responsiveness. Nevertheless, in recent years, it has become increasingly recognized that plants have genetically adapted their photosynthome in many ways in order to accommodate the specific environmental challenges. Studies of both intraspecific and interspecific variation repeatedly document divergent adaptation in photosynthetic traits (Guanter *et al.*, 2014; Nevado *et al.*, 2016). So far most of this research has ridden on the back of the genomics revolution and thus taken a reverse genetic approach, i.e. worked from the genotype towards the phenotype. Although photosynthetic processes are regularly implicated in the adaptation of plants to the environment, the precise phenotypic manifestation of these differences is rarely elucidated in terms of dynamic responses.

How can genetic and genomic approaches help to identify the cause of such variation in photosynthesis? To link the insights from population genetic studies in model plants such as in Nevado *et al.* (2016) to phenotypes, forward genetic approaches are ideal (Flood and Hancock, 2017). To succeed in identifying the causal loci, particularly those of small effect size, large numbers of genotypes (often >1000) should be phenotyped; such numbers also require high-throughput and high-quality phenomics. This supports the common statement that the genomics revolution has shifted the research bottleneck from genotyping to phenotyping (Flood *et al.*, 2016). Accurate phenotype data are essential for genetic mapping where an error rate of as little as 1–2 % can already result in spurious associations (James *et al*., 2013). Non-crop models have provided much of the early work, but the resources available for crop species are highly advanced, and crop species have now been used in phenotyping programmes for (steady-state) photosynthesis with mixed success (Driever *et al.*, 2014; Carmo-Silva *et al.*, 2017). It is again clear that large populations of plants need to be measured for quite complex and time-consuming traits such as gas exchange. In the field this is difficult, compounding the need for new advances in measurement technology. Recent work with elite lines of wheat, for example, has shown that variation in key (largely steady-state) traits exists but this does not link well with biomass and yield, demonstrating further the need for examination of dynamic traits (Driever *et al.*, 2014).

An important target to aid trait identification is understanding the mechanisms by which photosynthesis actually contributes to plant fitness, biomass and yield, and moreover how this varies with abiotic and biotic factors. This is not a simple task, for example in cereal plants the role of photosynthesis in forming yield can be dependent on developmental stage, and hence timing of measurement is important (Murchie and Reynolds, 2012). Therefore, understanding the range of selective and dynamic pressures that operate on photosynthesis in the field would greatly aid plant breeding programmes via the identification of new and dynamic traits and, importantly, which need measuring. Efforts to develop a big data approach to photosynthetic phenomics by recruiting many researchers into online cloud-based initiatives (Kuhlgert *et al.*, 2016) may be promising because not only can they assay many genotypes but they can also do so under the diverse conditions which plants experience in nature. When combined with fitness/yield data, the key photosynthetic phenotypes that constrain plant performance under naturally dynamic conditions can be identified. The caveat to this is the quality and consistency of in-field methodology. If succesful, this might be applied to traditional breeding or biotechnological solutions. All approaches could be made much more relevant when informed by models based on biological processes, as has recently been shown by altering expression levels of genes involved in photoprotection such as PsbS and those regulating the xanthophyll cycle (Kromdijk *et al.*, 2016). It follows that success could arise from the continued advancements in methodology (explained below) focused on the extraction of data describing dynamic traits across large numbers of genotypes in the field and informed by a good understanding of the biology that underpins yield components.

## PROXIMITY AND REMOTE SENSING – THE SPATIO-TEMPORAL VARIATIONS OF PHOTOSYNTHESIS FROM THE LEAF AND CANOPY TO THE (AGRO)ECOSYSTEM

Photosynthesis involves processes that span substantial temporal and spatial scales (Fig. 1). The absorption of photons in the photosynthetic pigments and the separation of excitons in the reaction centres happens on the time scales of femtoseconds and on the spatial scales of a few Ångstroms. On the other hand, photosynthesis is also quantified on the much larger spatial scale of canopies, fields and whole ecosystems, and temporal aggregates of photosynthetic carbon fixation are included in ecosystem models and used to predict global carbon budgets in times of global change.

Measurements of photosynthesis have historically been performed on single leaves using clip-on devices, methods which underpinned the great efforts to unravel and understand the molecular, biophysical and biochemical organization and functioning of the photosynthetic apparatus. In recent years, however, increasing scientific interest arose in measuring photosynthesis on larger scales to quantify local, regional and global carbon budgets and also to develop methods for fast and automated screening of photosynthetic traits for phenotyping approaches (Wohlfahrt and Gu, 2015). This inevitably confronted researchers with the challenge to measure photosynthesis under natural, i.e. fluctuating, conditions. Most of our scientific knowledge was obtained under controlled conditions in the laboratory and under a ‘steady state’ or a temporary ‘steady state’ in response to single variables such as light and CO_2_. In nature, photosynthesis, however, rarely operates under constant conditions but rather adapts to an ever-changing ‘stream’ of energy that also renders light availability in canopies spatially heterogeneous (Schurr *et al.*, 2006; Rascher and Nedbal, 2006). Temporal variability is translated into spatial heterogeneity, and we will discuss this interplay here.

In this context, chlorophyll fluorescence techniques are very important because they are non-contact and rapid, and have come to be a method of choice to understand the spatio-temporal dynamics of photosynthesis; hence the emphasis here. The classical pulse amplitude-modulated (PAM) approaches cannot always be considered, e.g. in remote applications. There are numerous reviews available that describe the principles and applications of chlorophyll fluorescence (e.g. Maxwell and Johnson, 2000; Baker, 2008; Murchie and Lawson, 2013).

In the following sub-sections, we review recent methods to measure photosynthesis remotely in the field and that are used to quantify photosynthesis on this larger scale, i.e. covering natural canopies, fields and even ecosystems by using aircraft and satellite platforms. In each case, we attempt to focus on the feature that allows the phenotyping of large numbers of plants at appropriate resolution, as explained in the previous section.

### Measuring photosynthesis from a distance using fluorescence transients

Pulse amplitude-modulated techniques brought PSII (photosystem II) chlorophyll fluorescence measurement from the lab to the field (Schreiber *et al.*, 1986; Murchie and Lawson, 2013; Porcar-Castell *et al.*, 2014). PAM methods use a saturating flash to measure minimum or steady-state fluorescence and maximum fluorescence, giving information on photochemical processes as well as the degree of photoprotective non-photochemical energy dissipation (NPQ). This method provides reliable data about photosynthetic performance (Schreiber *et al.*, 1986; Murchie and Lawson, 2013). As an alternative method, short sub-saturating flashes (a few at ≤1 μs) can be used to study fluorescence decay kinetics. Using sub-saturating flashes at a fast repetition rate triggers a light-induced fluorescence transient (LIFT), that allows the continuous recording of the fluorescence signal. These transients can be used to quantify the PAM parameters and additionally determine fluorescence parameters such as the photosystem cross-section of PSII or the time constants of electron transfer at PSII (Kolber *et al*., 1998, 2005).

For field approaches, the LIFT measurement approach has an enormous advantage; it can be used from some distance as the flashlets are of sub-saturating intensity. Based on laboratory experience, a first ‘remote sensing’ instrument was developed in 2001 and 2002 and first employed in the Biosphere 2 mesocosm (Ananyev *et al.*, 2005). Further technical development enabled this instrument to observe fluorescence signals from up to 50 m distance in a fast, non-invasive way to better understand photoprotection in arabidopsis (Kolber *et al.*, 2005), to monitor the dynamics of winter hardening (Pieruschka *et al*., 2007, 2014; Rascher and Pieruschka, 2008) and to monitor the seasonal dynamics of photosynthetic adaptation in different barley varieties (Raesch *et al.*, 2014). A new, lighter and more integrated LIFT instrument has been developed using light-emitting diodes (LEDs) at 470 nm wavelengths with maximal operating distance of a few metres (Osmond *et al.*, 2017; Wyber *et al.*, 2017). The nature of LIFT means that it could track dynamic shifts in PSII efficiency and NPQ quite easily in a remote setting and at high spatial scale, which would be a significant advance. In terms of ‘mapping’ fluorescence across plant canopies and accounting for spatial heterogeneity, the diameter of the measuring beam may be critical. This can be quite high (up to 10 cm) when operating from a distance but reduced in the LED version to 3 cm when measuring from 60 cm distance.

### Measuring and mapping sun-induced fluorescence emission – a new approach to quantify photosynthesis across huge scales

The LIFT measurement approach helped to overcome the limitations of the clip-on PAM devices, and first canopy screening experiments were facilitated. The next scaling would target a mapping of fluorescence on the field, ecosystem or even continental scale sun-induced fluorescence. The measurement concept takes advantage of solar and atmospheric absorption lines in which the incoming irradiance is greatly reduced. In these lines, the emitted weak fluorescence signal can be detected passively by using high-resolution spectrometers (Plascyk and Gabriel, 1975; Carter *et al.*, 1990; Moya *et al.*, 2004). In recent years, this measurement principle was used for remote sensing of vegetation (for reviews, see Malenovský *et al.*, 2009; Meroni *et al.*, 2009) and to detect vegetation stress (for a review, see Ač *et al.*, 2015).

The rapid technical development of high-resolution spectrometers in the past years further promoted the scientific exploitation of the sun-induced fluorescence (SIF) signal for photosynthetic activity. Thermoregulated and carefully arranged point spectrometers were used to record diurnal and seasonal time series of canopy fluorescence (Rossini *et al.*, 2010; Meroni *et al.*, 2011). Newer generations of these instruments were used to measure and compare canopy fluorescence across various ecosystems (Rossini *et al.*, 2015) and to better understand the contribution of structural and functional effects in ecosystem adaptation to nitrogen level (Migliavacca *et al.*, 2017). The measurement principle that was developed for point spectrometers could recently also be applied to ground-based imaging spectrometers (Pinto *et al.*, 2016) as well as to a high-resolution airborne imaging sensor HyPlant (Rascher *et al.*, 2015; Rossini *et al.*, 2015; Simmer *et al.*, 2015; Wieneke *et al.*, 2016). Recently, it was also possible to retrieve the relatively weak fluorescence signal from existing atmospheric satellites by fine tuning data acquisition and data retrieval (Frankenberg *et al.*, 2011; Joiner *et al*., 2011, 2013; Guanter *et al*., 2012, 2014). Following spatial and temporal averaging to retrieve the relatively weak signal, the novel information content of this new remote sensing signal and its application within agriculture could clearly be demonstrated by, for example, detecting photosynthetic hot-spots within the corn-belt of the USA or by describing the disconnection between canopy greenness and photosynthetic activity during the dry period in Australia (Guanter *et al.*, 2014). It is likely to have applications in tracking photosynthetic activity over wide spatio-temporal scales (Yang *et al.*, 2015). The huge scales over which SIF is measured and its low resolution will define its application in crop science and crop improvement. It is unclear as yet whether the resolution of the SIF signal into components of photosynthesis (such as photochemical or non-photochemical) is possible, but this would overcome some of the difficulties of conventional fluorescence imaging (see below).

Significant challenges remain (to measurement of dynamic photosynthesis) but advanced non-linear retrieval methods such as spectral fitting of the whole high-resolution spectrum have shown promising results (Cogliati *et al.*, 2015). Excitingly, this will also be the basis for a future dedicated satellite mission FLEX, which will be launched in 2022 as the Eighth Earth Explorer from the ESA and which will deliver high-resolution global maps of SIF (Drusch *et al.*, 2017).

### Dynamic thermal imaging to assess stomatal behaviour/kinetics

The ability to assess the spatially and temporally variable dynamics of other physiological parameters that directly affect or are affected by photosynthetic processes is key to understanding the mechanistic bases of photosynthetic processes in the field environment (Matthews *et al.*, 2018). For example, both photosynthesis and stomata respond to changes in light intensity; however, stomatal responses are an order of magnitude slower than photosynthetic responses (Kirschbaum and Pearcy, 1988; Tinoco-Ojanguren and Pearcy, 1993; Lawson and Weyers, 1999; Lawson *et al.*, 2010; McAusland *et al.*, 2016). Fluctuations in light through sun and shade flecks drive temporal and spatial dynamics in carbon gain and water loss (Barrada and Jones, 1996; Lawson and Weyers, 1999; Lawson and Blatt, 2014). Slow stomatal conductance (*g*_s_) responses to increasing light result in restriction of CO_2_ diffusion to match mesophyll demands for photosynthesis or slow stomatal closure when light decreases resulting in unnecessary water loss for no carbon gain (McAusland *et al.*, 2016). This leads to a disconnection between *g*_s_ and assimilation rate (Lawson *et al.*, 2012) and therefore plant water use efficiency (Lawson and Blatt, 2014) which is defined as the ratio of CO_2_ uptake relative to water lost. In addition, stomatal behaviour has important consequences for evaporative cooling and leaf temperature, nutrient uptake, translocation and plant water status.

**Fig. 2.**
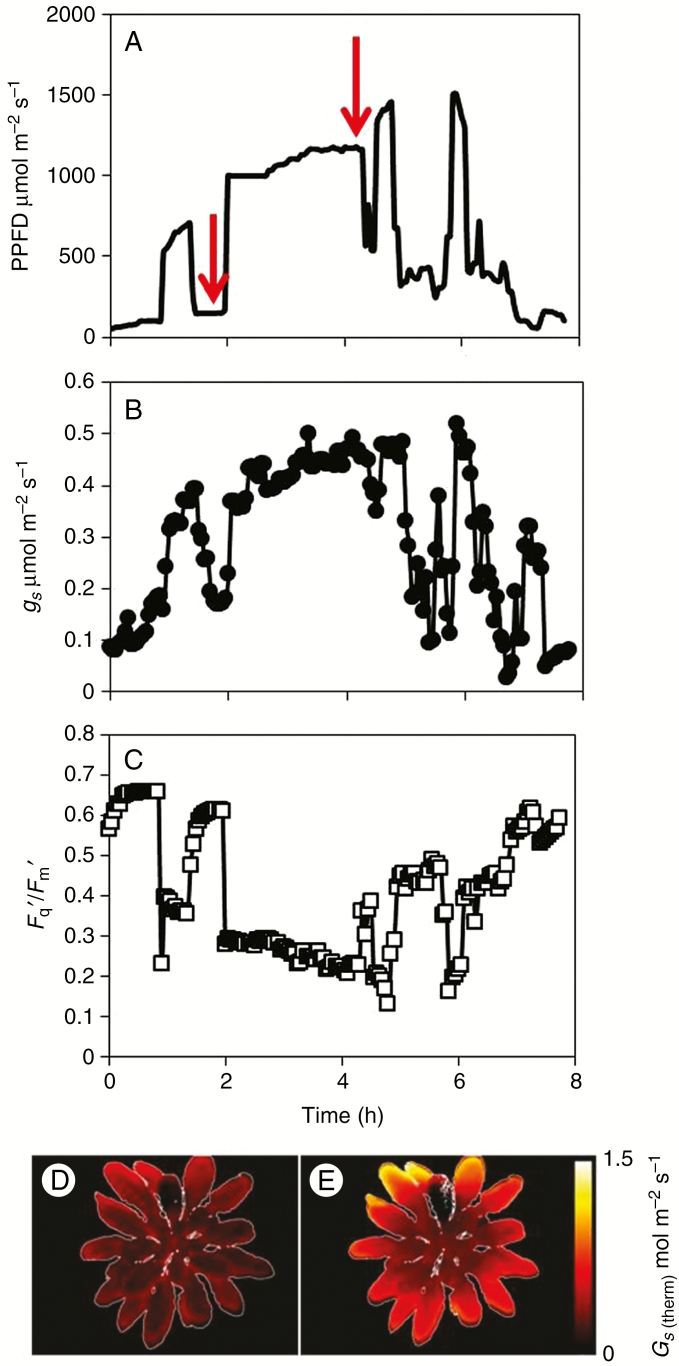
Diurnal light regimes (A) for measuring kinetics of stomatal conductance (*g*_s_) determined from thermography (B) and (C) photosynthetic efficiency (*F*_q_′/*F*_m_′) by chlorophyll fluorescence imaging in *Arabidopsis thaliana*. Two example images of stomatal conductance determined from images of leaf temperature (D and E) were captured at low and high light intensities (illustrated by the red arrow in (A). Apart from irradiance all other environment conditions were maintained constant (unpublished data of Vialet-Chabrand & Lawson).

Identifying genotypes, cultivars, accessions and species with more rapid stomatal responses that are synchronized with mesophyll photosynthetic rates could improve both photosynthesis (Lawson *et al.*, 2012; Matthews *et al*., 2017) and plant water use efficiency (Lawson and Blatt, 2014). The dynamic response of stomata or *g*_s_ to fluctuations in light intensity has been studied in several understorey forest-dwelling species, but relatively few reports have studied crop species (Chazdon and Pearcy, 1986; Chazdon, 1991; Tinoco-Ojanguren and Pearcy, 1993; Leakey *et al.*, 2005; McAusland *et al.*, 2016). Additionally, the majority of these studies have relied on examining stomatal kinetics using either porometry, which is notoriously noisy, or infrared gas exchange analysis which is time consuming. Thermography offers an alternative high-throughput phenotyping approach to assess stomatal behaviour (Omasa *et al.*, 1981; Hashimoto *et al.*, 1984; Wang *et al.*, 2004; Leinonen *et al.*, 2006; Jones *et al.*, 2009; McAusland *et al.*, 2016). Higher stomatal conductance leads to greater evaporative cooling of the leaf and a lowering of leaf temperature; as a result thermal imaging of leaf temperature can provide a convenient and reliable method for assessing stomatal behaviour. Thermal screens have been used successfully to identify a number of stomatal mutants (e.g. Merlot *et al.*, 2002; Wang *et al.*, 2004; Xie *et al.*, 2006). An important advance is that measurements of leaf temperature can also be converted to *g*_s_ using the basic energy balance equations (see Jones, 1999, 2004; Leinonen *et al.*, 2006). However, to date, the majority of these studies have relied on steady-state measurements both in the laboratory and in the field (Grant *et al.*, 2006). Recently, thermography has been shown to be a useful screening tool for examining dynamic stomatal behaviour in response to changing environmental cues and, in combination with measurements of photosynthesis via chlorophyll fluorescence imaging, it can be used to estimate plant water use efficiency (McAusland *et al*., 2013, 2015, 2016). For example, Fig. 2 shows *g*_s_ calculated from thermography from an arabidopsis plant subjected to a dynamic light regime Corresponding values of *F*_q_′/*F*_m_′ from chlorophyll fluorescence imaging illustrated that both *A* and *g*_s_ respond to the changes in light intensity, and, as expected, in opposite directions, but with different magnitudes of change. Such data can easily be used to determine the kinetics/speed of stomatal responses as well as provide a measure of the overall ‘steady-state’ *g*_s_ achieved under particular light levels. However, the negative aspect of using thermography to determine *g*_s_ is that the external/environmental conditions surrounding the leaf need to be known, as well as an estimate of the boundary layer resistance to water vapour (Jones, 1999; Jones *et al.*, 2002).

‘Wet’ and ‘dry’ reference standards that mimic the colour and shape of the leaf have been used to estimate the impact of changing environment conditions on temperature. The dry reference provides an infinite resistance to water vapour, whilst the wet provides a near-zero resistance to water vapour. These references standards are used to normalize the measured leaf temperature to the environmental conditions surrounding it, and it is assumed that these surfaces have the same radiative properties (Jones, 2004). Many different materials have been explored as reference materials; however, one of the best is using the leaf itself, with grease applied to both sides of the leaf providing a dry reference, while a leaf painted with a detergent–water mix provides a convenient wet reference (Guilioni *et al.*, 2008; McAusland *et al.*, 2013). Despite these complexities, under controlled conditions thermography provides an accurate and quantitative non-invasive tool for measuring spatial and temporal variation in *g*_s_, providing a rapid screen for stomatal dynamics that can be combined with other spectral signatures (such as chlorophyll fluorescence) to provide novel screening platforms such as plant water use efficiency (McAusland *et al*., 2013, 2016).

### 3-D analysis of photosynthesis and canopy photosynthesis dynamics

Canopy structure is a complex trait that needs to be optimized to account for the various trade-offs between light interception, light distribution and other field factors. An ideal canopy would result in a display of leaves that results in a maximum light interception and distributes photosynthetic activity effectively to enhance overall carbon gain per unit ground area. In reality, canopy architecture is highly variable (even among genotypes of the same species) and difficult to quantify. A high degree of self-shading is frequently observed, one function of which may be to compete effectively with weed growth, and the resulting density of foliage can hinder accurate 3-D analysis (Townsend *et al.*, 2018; Walker *et al.*, 2018). Canopy architecture is critical for photosynthesis because it defines the optimal leaf area index for the canopy, the linearity of the canopy–light photosynthesis relationship and the overall canopy photosynthetic rate (Murchie and Reynolds, 2012; Song *et al.*, 2013). For example, leaf angle is considered to be strongly linked to canopy photosynthetic rate, although this depends on growing environment (Hammer *et al.*, 2009). Therefore, it may be possible to improve canopy photosynthesis by tweaking architecture. However here we are primarily concerned with measuring photosynthesis *in situ* within such complex 3-D structures.

The 3-D architecture of a canopy creates a dynamic light environment. Solar movement and the movement of the canopy in wind creates fluctuations that are spatio-temporally highly complex and occur within sub-seconds to minutes to hours (Song *et al.*, 2013; Burgess *et al.*, 2016). Measurement of such light fluctuations with existing equipment would be difficult, but not impossible, since the numbers of sensors would be large and may themselves physically impede light transmission. Traditional canopy analysis uses parameters that are relatively easy to measure, e.g. leaf area index, fractional interception and canopy extinction coefficient. These are important because they permit a tractable means of mathematically linking light absorption with complex features of leaf angle and foliage density, but they do not provide knowledge of light fluctuations (Hirose, 2005).

Together with knowledge of 3-D canopy architecture, light fluctuations can be defined by light ray tracing techniques to predict photosynthetic responses (Song *et al.*, 2013). Many techniques for 3-D reconstruction of entire canopies have been published (e.g. Godin, 2000; Godin and Sinoquet, 2005; Watanabe *et al.*, 2005; Quan *et al.*, 2006; Wang *et al.*, 2008; Paulus *et al.*, 2014; Pound *et al.*, 2014; Gibbs *et al.*, 2017; Townsend *et al.*, 2018). With such models of 3-D architecture, and even canopy movement, it is possible to predict photosynthesis dynamics at high resolution (Burgess *et al.*, 2016). Techniques for 3-D reconstruction using, for example, laser- or RGB- (for red, green and blue light) based techniques will typically result in a 3-D point cloud that can be processed to generate 2-D leaf surfaces for downstream processes such as ray tracing that can accurately predict light fluctuations within the canopy (Pound *et al.*, 2014). The level of investment in infrastructure varies enormously: some automated techniques require large field installations (Hawkesford and Lorence, 2017; Virlet *et al.*, 2017), while others can be bought at low cost and operated manually or automatically (Pound *et al.*, 2014). A substantial issue is the density of the canopy and the problem of occlusion, meaning, for example, that it is usually only possible to visualize completely the ‘top’ or projected surface area (that excludes overlapped leaves) of a mature field canopy without as yet unavailable techniques such as field computed tomography (CT) scanning. The internal arrangement of leaves may therefore not be visible. This can be overcome by removing plants and scanning (Burgess *et al.*, 2015). Some approaches have partially overcome this imaging problem (Busemeyer *et al.*, 2013; Großkinsky *et al.*, 2015).

Attaining high-resolution 3-D reconstructions of canopies may be an important first step. This is largely because it is not currently physically possible to measure/monitor photosynthesis at every point within a large and complex canopy. The most common approach is to use portable gas exchange and chlorophyll fluorescence, either at fixed points during the day or as part of a diurnal, to parameterize canopy photosynthesis models in combination with the 3-D reconstruction or an approximation. Canopy photosynthesis modelling is a relatively common technique (Zhu *et al.*, 2012; Song *et al.*, 2017; Wang *et al.*, 2017). This can be done to great effect and has on occasions been validated using the more difficult whole-canopy gas exchange chambers (Song *et al.*, 2016). Long-term chlorophyll fluorescence monitoring techniques are available (Porcar-Castell *et al.*, 2012; Hubbart *et al.*, 2018) and provide high-resolution information on photoprotection and photosynthesis data, but the sensors are large and it is only possible to monitor a small proportion of the leaf surface.

While chlorophyll fluorescence imaging would seem to be a logical step (see other sections in this review), PAM fluorescence suffers from the problem of not being able to cope with great depth or issues such as leaf curvature due to the need to illuminate the leaf evenly, dark adapt the leaf and provide an even saturating flash (Murchie and Lawson, 2013). Previous requirements for dark adaptation of material that would preclude the ability to measure some dynamic processes such as NPQ has been partly overcome with the development of NPQ_(T)_ which does not require dark adaptation (Tietz *et al.*, 2017). *Arabidopsis thaliana* has a flat rosette canopy and is relatively simple to scan as a 2-D object. Early in plant development when canopy complexity and leaf area index are relatively low, crops such as wheat may be able to be treated as a 2-D surface with some systems. There is currently no way to measure photosynthesis, *in situ*, in all points of the (occluded) canopy. The best strategy may be to use a large number of monitoring fluorometers (also possible with multiple gas exchange chambers) scattered among a large canopy so that the devices do not impede light transmission.

Given the increasing importance of complex light patterns within plant canopies and the impact they have on biomass and yield (Burgess *et al.*, 2016; Kromdijk *et al.*, 2016; Townsend *et al.*, 2018), it is critical to continue to find new ways of visualizing canopies in three dimensions and measuring photosynthetic dynamics accurately across all (or substantial parts) leaf areas over long time periods. It is fair to say that we have not yet achieved this and for the foreseeable future we may need to rely on 3-D reconstruction combined with modelling and photosynthesis measured/imaged on canopy parts only. Importantly, there have been recent advances in the inclusion of dynamic processes in photosynthesis modelling at the leaf level that may lend themselves to scaling to the canopy (Pearcy *et al.*, 1997; Muller, 2011; Zhu *et al*., 2012, 2013; Kaiser *et al*., 2015, 2018). We may see robotic technology capable of highly mobile, discrete in-canopy measurement of architecture and photosynthesis simultaneously.

### Affordable high-resolution field phenotyping: problems and opportunities

As highlighted above, many key points regarding the factors that contribute to plant photosynthesis and crop yield often come from a body of knowledge based on controlled experiments with a high frequency of measurements using state-of-the-art and expensive sensors (Fiorani and Schurr, 2013; Cabrera-Bosquet *et al.*, 2016; Hawkesford and Lorence, 2017; Kirchgessner *et al.*, 2017; Virlet *et al.*, 2017). However, when measuring photosynthetic performance under field conditions in a high-throughput manner, it is difficult to capture complex dynamic information. It is evident that a trade-off exists between throughput in data acquisition and the precision of the information gathered. With this premise in mind, we may discern the most efficient and effective techniques for field phenotyping towards measuring photosynthetic performance. A practical dilemma often encountered is the optimal selection of instrumentation for budget and field conditions, allowing the precise timing of data acquisition, and the best approach for data analysis (White *et al.*, 2012; Araus and Cairns, 2014). More is not always better in field phenotyping, as time is limited and a focus purely on quantity results in a loss of quality. Easily attainable high-spatial resolution image data using RGB broadband visible light reflectance may provide more meaningful data for quantifying biomass/growth and photosynthetic pigments compared with lower spatial resolution narrowband multispectral VNIR (visible plus near-infrared light) measurements, and measuring both may result in data overlap (Gracia-Romero *et al.*, 2017; Kefauver *et al.*, 2017). An efficient and focused approach on specific traits of interest will lead to better quality data and results, with the desired levels of high throughput (Tambussi *et al.*, 2005, 2007; Kefauver *et al.*, 2015; Zhou *et al.*, 2015). Here we consider the state of the art in such techniques and then how applicable they may be to ‘dynamic’ phenotyping.

Time-consuming measurements such as carbon assimilation parameters (through an infrared gas analyser, IRGA) that directly measure carbon assimilation can be very insightful and certainly can capture dynamic changes in photosynthesis and photoprotection in a lot of detail (Kromdjik *et al*., 2016) but are currently not high throughput and require expensive instruments. Even portable porometers, which offer a more convenient and higher throughput alternative compared with IRGA, are not a feasible alternative when large-scale phenotyping is required. Other alternatives include portable spectroradiometers with active sensors, but they do not measure photosynthesis directly. These can be used to assess total photosynthetic surface area, for example through vegetation indexes, such as the normalized difference vegetation index (NDVI). Leaf pigment meters use absorbance for chlorophyll content and other pigments, such as anthocyanins and flavonoids, that are indicative in the photosynthetic responses to stress conditions. Infrared thermometers can measure canopy temperature as a surrogate of transpiration (see above) although they have some disadvantages, e.g. in wind and on cool days. These devices provide meaningful and high-throughput data on plant physiological conditions related to plant vigour, photosynthetic capacity and photosynthetic efficiency, as well as responses to different categories of abiotic and biotic stresses, but will not capture complex photosynthetic dynamics (Prasanna *et al.*, 2013; Winterhalter *et al.*, 2013; Araus and Cairns, 2014). The use of these approaches for breeding is not new. Infrared thermometers and portable spectroradiometers that provide proxy measurements may be considered as scientifically reliable, providing direct measurements of pigment content or leaf temperature (as proxies of potential photosynthetic and stomatal conductance, respectively). The widespread use of these traditional techniques has largely reached its limit in producing new scientific insight for phenotyping, and thus new techniques adapted from the field of remote sensing are being applied more proximally and at higher resolution (Fiorani *et al.*, 2012; Fiorani and Schurr, 2013).

Thermal imaging may be granted separate consideration in terms of the importance of the effects of plant temperature on the dynamic processes of plant photosynthesis and the challenges presented in its measurement. Infrared thermometers, in spite of low cost and easy use, have not been widely adopted as phenotyping tools to assess abiotic stresses such as water, heat or salinity stress. Thermal cameras represent an alternative, but so far the cost has been prohibitive: recent developments have substantially reduced both size and cost. Thermal measurements and thermal image acquisition for plant photosynthesis phenotyping in the field comes with its own unique sets of problems and opportunities. As described above for controlled environments, the key issue is that plant temperature is a very dynamic variable with a high impact on plant photosynthesis, including photosynthetic capacity, photosynthetic efficiency and water use. Temperature measurements across numerous phenotyping plots should be acquired as quickly and precisely as possible, with added benefits from multiple acquisitions per day and under different meteorological conditions for optimal insight (Gonzalez-Dugo *et al.*, 2015). For thermal imaging, the case can be made for significant benefits from acquisition from an aerial platform with a greater capacity for near-simultaneous measurement across plots (Zarco-Tejada *et al.*, 2012; Gonzalez-Dugo *et al.*, 2015). Thermal imaging also has a greater potential for measuring dynamic changes in gas exchange properties than, for example, RGB, but this has not been fully realized in the field (see section on imaging above).

Similarly, the analysis of the stable isotope signatures in plant matter can provide key insights into cumulative photosynthetic activity and has been successful in breeding for water use efficiency (Farquhar *et al.*, 1989; Lopes *et al.*, 2004; Sanchez-Bragado *et al.*, 2014). However, it depends on fairly rapid sampling, can be time consuming in preparation and analysis, is costly for a large number of samples and does not provide insight into the mechanism of specific photosynthesis dynamics but rather their accumulated impact over time. Nevertheless, the use of near-infrared reflectance spectroscopy (NIRS) may represent an alternative (Cabrera-Bosquet *et al.*, 2012; Araus and Cairns, 2014).

### Broadband visible light reflectance at high spatial resolution from RGB cameras

From studies of high-resolution field spectroscopy and the spatial dimension added in hyperspectral or imaging spectroscopy work, we can identify a suite of targeted multispectral vegetation reflectance indices that indicate specific plant physiological components related to cumulative and more dynamic photosynthetic processes (Filella *et al.*, 1996; Gitelson *et al.*, 2002; Ustin *et al.*, 2009; Lobos *et al.*, 2014). Similarly, very high spatial resolution image data take advantage of the relatively low cost commercial sensors that provide very high-resolution visible light (RGB) digital images. These same cameras can also be modified (mRGB), albeit with some additional need for calibration (Rasmussen *et al.*, 2016; Berra *et al.*, 2017) to capture near-infrared and red-edge light for capturing high spatial resolution spectral indices, such as NDVI or the normalized difference red-edge index (NDREI). Furthermore, these commercial RGB and mRGB cameras cost a fraction of multispectral scientific instruments, may provide equally meaningful data toward plant photosynthesis phenotyping in the field and are equally adaptable to unmanned aerial vehicle (UAV) platforms (Tattaris *et al.*, 2016). Additionally, the high spatial resolution of these commercial cameras may provide precise 3-D reconstructions used to estimate plant spatial dimension details such as height, biomass and plant architecture (see earlier in this review) and even the possibility of segmentation and counting of individual plant components, such as fruit, wheat heads, maize ears and other important components related to yield prediction (Cointault *et al.*, 2008; Bulanon *et al.*, 2009; Patel *et al.*, 2011).

Lower cost and accessible RGB and mRGB cameras as broadband measurements in VNIR light reflectance may offer insights into the dynamic processes of plant growth at the scale of days and weeks, to produce, for example, detailed growth curves and phenological stage assessments. Currently they do not provide information on dynamic responses of photosynthesis over time scales of seconds and minutes; however, the closest method of this type could be the spectral reflectance indices such as PRI (photochemical reflectance index) or the similar CCI (chlorophyll/carotenoid index), which have not yet been widely used for this purpose (Gamon *et al.*, 1992, 2016; Gitelson *et al.*, 2017). Spectral indices have the potential to indicate changes in biochemical composition, but only a limited number have the potential to indicate dynamics on a fine scale, depending on their biological origin. For example, if the PRI signal is influenced by shifts in de-epoxidation of the xanthophyll cycle then it has the potential to do this (Alonso *et al.*, 2017). However, it is debatable whether spectral reflectance, especially when considering the more narrowband scientific spectrometers or imaging sensors needed for measuring, for example, the PRI and CCI, could be considered low cost. However, when considering sensors that were developed primarily for commercial and aesthetic image acquisition, we must be careful when applying them for scientific purposes. This includes standardized and careful planning in data acquisition, calibration, processing and validation. The continued development of such accessible methods deserves continued attention.

**Fig. 3.**
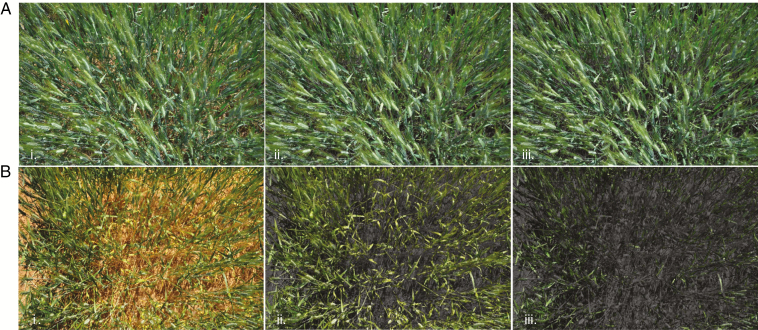
Irrigated (A) and rainfed (B) wheat phenotyping trial plots showing (i.) original RGB image, (ii.) GA (green area: hue 60–120) and (iii.) GGA (greener green area: hue 80–120) results from the Breedpix 0.2 portion of the FIJI plugin CIMMYT Maize Scanner. https://github.com/sckefauver/CIMMYT.

The most limiting time factor is often during data capture in the field. Nevertheless, image control data at the time of image acquisition are necessary and include images of calibration panels for white balance, colour and spatial distortion effects (Lebourgeois *et al.*, 2008; Rabatel *et al.*, 2011; Berra *et al.*, 2017). Improved processing of the calibrated images enables more consistent and accurate results. One example, in the case of calculating the common green pixel indexes GA (green area) and GGA (greener area) as done in the Breedpix 0.2 suite of indices (Casadesús *et al.*, 2007; Casadesús and Villegas, 2014), is the use of alternate colour spaces for providing some minor calibrations extracting the green pixel area within an image scene. The benefit of using hue, saturation and intensity (HSI) colour space is that hue represents one axis of the colour value separate from the illumination intensity and colour saturation components of the image. Segmentation based on green pixel values from the ‘Hue’ channel provides more consistent results compared with a direct extraction from green (Fig. 3). The use of normalized index calculations using an RGB or near-infrared-modified RGB camera image as a three waveband multispectral sensor may also result in more consistent and high-quality results that provide some internal calibration against illumination effects (Vogelmann *et al.*, 1993; Hunt *et al*., 2011, 2012; Li *et al.*, 2014; Kefauver *et al.*, 2015; Vergara-Diaz *et al.*, 2015; Zhou *et al.*, 2015; Kira *et al.*, 2016; Berra *et al.*, 2017).

Through standardized acquisition, calibration and processing, the combination of image analysis techniques either on field or UAV platforms may offer an ideal combination of efficient and cost-effective image acquisition for photosynthesis phenotyping providing data with both high spatial and temporal resolution, off the shelf sensors and modified digital cameras at a fraction of the cost of scientifically developed sensor systems. The next step is the development and implementation of such methods to capture dynamic photosynthesis over a scale of seconds, minutes and hours.

## BRACING PLANTS FOR CHANGE: THE DISTANCE BETWEEN THE STABLE LABORATORY AND THE DYNAMIC FIELD

When developing a phenotyping system, an important consideration is whether or not the environment should be controlled. Controlled environments have many experimental advantages in that they allow for a systematic testing of different environmental variables without having the confounding effects of co-varying environmental parameters. However, it is recognized that growing and measuring plants in controlled conditions does not necessarily translate to field responses. The terminology used in a recent review is appropriate: ‘pampered inside, pestered outside’ (Poorter *et al.*, 2016). This meta-analysis found only a moderate correlation between lab and field phenotypic data, and suggested that differences in light levels and planting density are important.

Perhaps field phenotyping systems should be prioritized because they have high capacity and low cost per unit area, and measure ‘real-world’ phenotypes that will provide the correct conditions for crop yield components. However, environmental conditions may vary, making several sites a necessity. Ideally one would use both approaches, i.e. disentangle subtle phenotypic responses in a controlled setting, perhaps in a model species, and validate relevance in a field setting, leading to genetic analysis and breeding. The species under study will to a large extent determine this.

A controlled environmental set-up is of most relevance when the physiological response to a specific environmental perturbance, for example light intensity, temperature, humidity or day length, is to be investigated. In such cases, keeping all other environmental conditions steady is key to assessing the effect the variable(s) of interest. An example of such a set-up is described by Flood *et al.* (2016) where the phenotyping and growth systems are integrated so that the act of phenotyping has a minimal effect, e.g. plant removal and movement. In some designs, plants are moved from a growth facility to a phenotyping station; this has the advantage that throughput is not limited by growth space, but comes at the cost of not having full environmental control; the act of moving the plants will always increase noise. Controlled environments can also offer possibilities to manipulate atmospheric concentrations of CO_2_ and O_2_ to mimic both past and future climates (Elliott-Kingston *et al.*, 2016) and accurate imitation of field conditions, for example the use of LED lighting can alter both intensity and spectral quality at a rate that matches field conditions (Vialet-Chabrand *et al.*, 2017; Matthews *et al.*, 2018). This shows how the uncoupling of environmental factors under realistic field-like environments may become a feasible route for achieving the lab to field connection.

## CONCLUSION AND PERSPECTIVES

Photosynthetic phenomics has now acheived the status of being able to conduct forward genetic screens in diverse species, which, when combined with genomics, should allow the identification of both the genetic and phenotypic changes which have facilitated photosynthetic adaptation to diverse environments. Such knowledge will prove essential to physiologists, ecologists and evolutionary biologists studying how plants adapt to various environments, and to conservationists and plant breeders aiming to facilitate wild or cultivated species to adjust to global climate change. As such, the phenomics of photosynthesis of large populations of crop species and of model plant species is of fundamental importance and is backed up by the enormous investment in both field and laboratory technology (Hawkesford and Lorence, 2017).

This review article concludes that we do not yet have the full capability for automated high-throughput phenotyping of all complex but essential photosynthetic traits, namely the efficiency of responses of photosynthesis to rapid changes in the environment. This conundrum is compounded by the fact that it would be beneficial to assay photosynthesis in the field where the environment is highly variable. One solution is to exploit the advances in controlled-environment technology where larger spaces that mimic the natural environment can be constructed and phenotyping technology can be integrated and advanced enough to measure dynamic traits on multiple plants.

Crop improvement strategies have advanced substantially since the dawn of the genomics revolution. Genomic selection is poised to improve complex traits such as dynamic photosynthesis, thus allowing maximal use of natural genetic variation present, and modern genome editing techniques will allow for novel phenotypic adjustments not present in the germplasm. For these improvement strategies, photosynthetic phenomics will play a key role acting as an essential catalyst, providing both the data necessary for them to work (genomic selection) and the data necessary to validate the most successful combinations of alleles (be they natural or edited) in diverse settings.
